# Polymorphisms of TNF-*α* -308 G/A and IL-8 -251 T/A Genes Associated with Urothelial Carcinoma: A Case-Control Study

**DOI:** 10.1155/2018/3148137

**Published:** 2018-05-21

**Authors:** Chia-Chang Wu, Yung-Kai Huang, Chao-Yuan Huang, Horng-Sheng Shiue, Yeong-Shiau Pu, Chien-Tien Su, Ying-Chin Lin, Yu-Mei Hsueh

**Affiliations:** ^1^School of Public Health, College of Public Health and Nutrition, Taipei Medical University, Taipei 110, Taiwan; ^2^Department of Urology, School of Medicine, College of Medicine, Taipei Medical University, Taipei 110, Taiwan; ^3^Department of Urology, Shuang Ho Hospital, Taipei Medical University, New Taipei City 235, Taiwan; ^4^School of Oral Hygiene, College of Oral Medicine, Taipei Medical University, Taipei 110, Taiwan; ^5^Department of Urology, National Taiwan University Hospital, College of Medicine, National Taiwan University, Taipei 100, Taiwan; ^6^Department of Chinese Medicine, Chang Gung Memorial Hospital and Chang Gung University, College of Medicine, Taoyuan 333, Taiwan; ^7^Department of Family Medicine, Taipei Medical University Hospital, Taipei Medical University, Taipei 110, Taiwan; ^8^Department of Family Medicine, School of Medicine, College of Medicine, Taipei Medical University, Taipei 110, Taiwan; ^9^Department of Health Examination, Wan Fang Hospital, Taipei Medical University, Taipei 116, Taiwan; ^10^Department of Family Medicine, Shuang Ho Hospital, Taipei Medical University, New Taipei City 235, Taiwan; ^11^Department of Public Health, School of Medicine, College of Medicine, Taipei Medical University, Taipei 110, Taiwan

## Abstract

Cigarette smoking and exposure to environmental tobacco smoke are well-known risk factors for urothelial carcinoma (UC). We conducted a hospital-based case-control study involving 287 UC cases and 574 cancer-free controls to investigate the joint effects of cigarette smoking and polymorphisms of inflammatory genes on UC risk. Tumor necrosis factor alpha (TNF-*α*)* -*308 G/A and interleukin-8 (IL-8) -251 T/A polymorphisms were determined using a polymerase chain reaction-restriction fragment length polymorphism method. People who had ever smoked and those who were exposed to environmental tobacco smoke had significantly increased UC odds ratios (ORs) of 1.65 and 1.68, respectively. Participants who had smoked more than 18 pack-years had a significantly increased UC OR of 2.64. People who had ever smoked and who carried the A/A genotype of the* TNF-α -*308 G/A polymorphism had a significantly higher UC OR (10.25) compared to people who had never smoked and who carried the G/G or G/A genotype. In addition, people who had ever smoked and who carried the* IL-8* -251 T/T genotype had a significantly increased UC OR (3.08) compared to people who had never smoked and who carried the T/A or A/A genotype. In a combined analysis of three major risk factors (cumulative cigarette smoking, the* TNF-α -*308 A/A genotype, and the* IL-8* -251 T/T genotype), subjects with any one, any two, and all three risk factors experienced significantly increased UC ORs of 1.55, 2.89, and 3.77, respectively, compared to individuals with none of the risk factors.* Conclusions*. Our results indicate that the combined effects of cumulative cigarette exposure and the* TNF-α -*308 A/A genotype and/or the* IL-8* -251 T/T genotype on UC OR showed a significant dose-response relationship.

## 1. Introduction

The bladder is the most common site of urothelial carcinoma (UC), a multifactorial malignancy influenced by exogenous exposure to environmental risk factors and molecular variations in metabolism-related genes [1]. Cigarette smoking is the major risk factor for bladder cancer, and epidemiological studies have indicated that cigarette smokers have a 2~4-fold increased risk of bladder cancer [2]. Cigarettes contain approximately 60 chemical carcinogens, including polycyclic aromatic hydrocarbons, aromatic amines, and N-nitroso compounds, which are associated with the development of bladder cancer [3]. Cigarette smoke contains free radicals and induces oxidative stress, which is associated with the development of bladder cancer [4, 5].

Oxidative stress can induce proinflammatory cytokines such as tumor necrosis factor alpha (TNF-*α*) and interleukin-8 (IL-8), which are involved in the development of various malignancies [6, 7]. Exposure to cigarette smoke can enhance TNF-*α* expression through upregulating the activator protein, AP-1 [8]. TNF-*α* is involved in the immune response during the intravesical instillation of bacillus Calmette-Guerin [9]. Previous studies reported that cigarette smoking can induce inflammation that is characterized by increased levels of cytokines such as TNF-*α* and IL-8 [10, 11]. IL-8 is a proinflammatory chemokine that is secreted by various cell types. Studies showed the chronic inflammation is involved in various tumorigenesis steps. TNF-*α* initiates signaling pathways that activate proinflammatory gene expression via the transcription factor; nuclear factor-kappa B (NF-*κ*B) is also produced by tumors and acts as an endogenous tumor promoter. Cytokines, like IL-6 and IL-8, are also involved in transformation and angiogenesis, respectively [12]. Environmental-genetic associations with tobacco smoke combined with genetic polymorphisms of inflammation-related genes provide additional bladder cancer risk.

The polymorphism of* TNF-α* gene (-308 G/A, rs1800629) is located in promoter and has functional impact on the TNF-*α* expression [13–15]. Compared to SNPs of TNF-*α*, -308 G/A polymorphism was investigated and found to be related to several cancers, including breast, gastric, and bladder cancers [13, 16–18]. However, results from previous studies were inconsistent. The* IL-8* gene is located on chromosome 4q13-q21, and several polymorphisms were identified in this gene [19]. A common polymorphism at the promoter region (-251 T/A, rs4073) was reported to influence the transcriptional activity of* IL-8*, and it was identified to be associated with various cancer risks in Asian group [6]. Therefore, these findings raise the possibility that genetic variations in the* TNF-α* and* IL-8* genes may modify the risk of UC.

To investigate the effects of* TNF-α -*308 G/A and* IL-8* -251 T/A polymorphisms on the UC risk, we conducted a hospital-based case-control study. We also examined the combined effects of cumulative cigarette exposure, the* TNF-α -*308 A/A genotype, and the* IL-8* -251 T/T genotype on UC risk.

## 2. Material and Methods

### 2.1. Study Population

This study involved 861 participants enrolled from the Departments of Urology and those undergoing a general health examination at National Taiwan University Hospital, Taipei Medical University Hospital, and Taipei Municipal Wan Fang Hospital between September 2002 and May 2009. In total, 287 UC cases were included (with a mean age of 62.95 ± 13.62 years). Each case was diagnosed with histopathological confirmation, which was performed using routine urological practices and verified by board-certified pathologists. In total, 574 age- and gender-matched cancer-free controls (with a mean age of 62.56 ± 13.50 years) were recruited from a hospital-based pool. All participants provided informed consent before the questionnaire interview and biospecimen collection. The Research Ethics Committee of National Taiwan University Hospital (Taipei, Taiwan) approved this study, and this study complied with the World Medical Association* Declaration of Helsinki*.

### 2.2. Questionnaire Interview and Biospecimen Collection

Well-trained investigators interviewed each patient using a structured questionnaire. Information collected by the questionnaire included demographic characteristics and lifestyle habits such as cigarette smoking, secondhand smoke exposure, betel nut chewing, alcohol, tea, and coffee consumption, exposure to occupational and environmental carcinogens such as pesticides, and a family history of disease. Study subjects who had smoked more than 100 cigarettes during their lifetime were regarded as people who had ever smoked, while those who had smoked fewer than 100 cigarettes were defined as people who had never smoked. Environmental tobacco smoke (secondhand smoke exposure) was assessed through a questionnaire asking participants whether anyone had ever smoked around them. A 6~8-ml sample of venous blood was drawn from each participant for genotype determination.

### 2.3. Genotyping of the* TNF-α -*308 G/A and* IL-8* -251 T/A Polymorphisms

**Figure 1:**
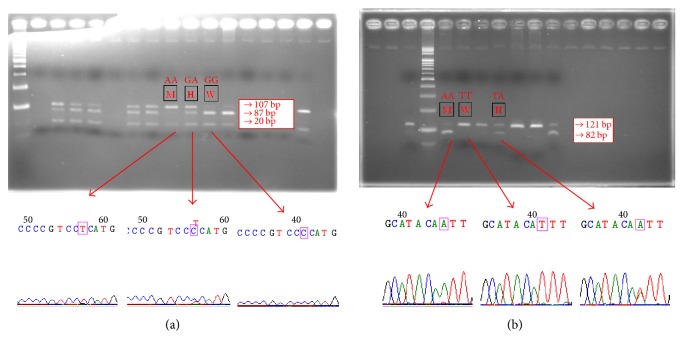
PCR-RFLP genotyping and sequencing of* TNF-α -*308 G/A and* IL*-8 -251 T/A. (a) Genotyping of* TNF-α -*308 G/A. Arrows indicated location of AA, GA, and GG. (b) Genotyping of* IL*-8 -251 T/A. Arrows indicated location of AA, TT, and TA.

Genomic DNA was extracted from the buffy coat of peripheral blood using standard methods and stored at −80°C. Genotyping was completed using a polymerase chain reaction-restriction fragment length polymorphism (PCR-RFLP) method modified from a study by Duarte et al. [20]. Briefly, the following primer sets were designed for the* TNF-α -*308 G/A polymorphism: 5′-TCCTCCCTGCTCCGATTCCG-3′ (sense) and 5′-AGG CAATAGGTTTTGAGGGCCAT-3′ (antisense). The thermal PCR conditions were as follows: one cycle at 95°C for 5 min; 35 cycles of 95°C for 30 s, 58°C for 30 s, and 72°C for 45 s; and a final extension at 72°C for 10 min. After complete digestion with the NcoI restriction enzyme, the resulting DNA fragments, which represented the* TNF-α -*308 G/A polymorphism, were determined (G/G: 87 and 20 bp; G/A: 107, 87, and 20 bp; and A/A: 107 bp). PCR-RFLP genotyping and sequencing of* TNF-α -*308 G/A are shown in Figure 1(a). For the* IL*-8 -251 T/A polymorphism, the following primers were used: 5′-ATCTTGTTCTAACACCTGCCACTC-3′ (sense) and 5′-TAAAATACTGAAGCTCCACAATTTGG-3′ (antisense) [21]. The PCR conditions were as follows: one cycle at 94°C for 5 min; 35 cycles of 94°C for 50 s, 61°C for 60 s, and 72°C for 55 s; and a final extension at 72°C for 5 min. The genotypes were determined after digestion with the MfeI restriction enzyme for the* IL*-8 -251 T/A polymorphism (T/T: 121 bp; T/A: 121, 82, and 39 bp; and A/A: 82 and 39 bp). PCR-RFLP genotyping and sequencing of* IL*-8 -251 T/A are shown in Figure 1(b). For quality control, genotyping was repeated on a random 10% of the samples.

### 2.4. Statistical Analysis

A goodness-of-fit Chi-squared test was used to test for Hardy-Weinberg equilibrium (HWE) among the cancer-free controls. Study subjects who had smoked more than 100 cigarettes during their lifetime were regarded as people who had ever smoked, while those who had smoked fewer than 100 cigarettes were defined as people who had never smoked. The pack-years of cigarette smoking were calculated using the formula: pack-years = (cigarettes per day ÷ 20) × (number of years smoked). Because of dependent variable (UC) is binary, logistic regression is used to assess the association between UC and related risk factors. The combined effects of cumulative cigarette exposure, the* TNF-α -*308 A/A genotype, and the* IL-8* -251 T/T genotype on the UC risk were estimated by odds ratios (ORs) and associated 95% confidence intervals (CIs) using a multivariate-adjusted logistic regression. All data were analyzed using the Statistical Analysis Software for Windows, ver. 9.2 (SAS Institute, Cary, NC).* p* values of < 0.05 were considered statistically significant.

## 3. Results

No significant differences were found in age or gender between the UC cases and cancer-free controls. Occasional alcohol drinkers had a significantly lower OR for UC than people who had never consumed alcohol. After adjusting for age, gender, educational level, alcohol consumption, and other risk factors, people who had ever smoked had a significantly higher UC OR of 1.65 (95% CI: 1.19~2.27) compared to people who had never smoked. Individuals who were exposed to environmental tobacco smoke had a significantly higher UC OR (1.68, 95% CI: 1.22~2.30) than those with no exposure. Frequencies of the* TNF-α -*308 A/A genotype and the* IL-8* -251 T/T genotype among controls fit HWE (*χ*^2^ = 0.99, *p* > 0.05; *χ*^2^ = 1.27, *p* > 0.05, resp.). The genotype distribution of the control did not show significant difference from the Hardy-Weinberg equilibrium values. Subjects who carried the* TNF-α*-308 A/A genotype had a significantly increased OR for UC (3.56, 95% CI: 1.03~12.28) compared to those with the G/G or G/A genotype. Subjects who carried the A/A genotype of the* IL-8* -251 T/A polymorphism had a significantly lower OR for UC (0.52, 95% CI: 0.27~0.99) than those carrying the T/T genotype (data not shown).

After stratification by the* TNF-α -*308 G/A and* IL-8* -251 T/A genotypes, individual characteristics, including age, gender, educational level, cigarette smoking, environmental tobacco smoke exposure, and alcohol consumption, displayed no significantly different distributions in either of the polymorphism strata (Table 1). In terms of the effect of the cigarette smoking profile on the OR for UC, we found significantly increased ORs (95% CI) for UC of 2.33 (1.52~3.55), 2.74 (1.77~4.24), and 2.64 (1.75~3.99) for subjects who had smoked more than 1 pack per day, individuals who had smoked for more than 31 years, and those who had smoked more than 18 pack-years, respectively (Table 2).

**Figure 2:**
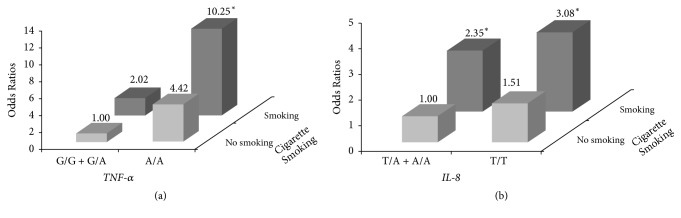
Joint effects of (a) the tumor necrosis factor *α* (TNF-*α*) genotype and cigarette smoking and (b) the interleukin 8 (IL-8) genotype and cigarette smoking on the urothelial carcinoma (UC) risk after adjusting for age, gender, educational level, and alcohol consumption. ^*∗*^*p* < 0.05.

The joint effects of cigarette smoking, the* TNF-α -*308 A/A genotype, and the* IL-8* -251 T/T genotype on the OR for UC after multivariate adjustment are shown in Figure 2. People who had ever smoked and who carried the A/A genotype of the* TNF-α -*308 G/A polymorphism had a significantly higher UC OR of 10.25 than people who had never smoked and who carried the G/G or G/A genotype. In addition, people who had ever smoked who carried the T/T genotype of the* IL-8* -251 T/A polymorphism had a significantly increased UC OR of 3.08 compared to those who carried the T/A or A/A genotype.

Furthermore, we included three major risk factors (cumulative cigarette smoking, the* TNF-α -*308 A/A genotype, and the* IL-8* -251 T/T genotype) in a combined analysis (Table 3). Compared to individuals who did had none of these risk factors, significantly increased ORs (95% CI) for UC were 1.55 (1.03~2.35), 2.89 (1.70~4.93), and 3.77 (2.16~6.56) for study subjects with any one, any two, and all risk factors, respectively; these risk factors showed a significant dose-response relationship (*p* for trend < 0.0001).

## 4. Discussion

In this study, the risk of UC in smokers was 2~2.5-fold compared to nonsmokers. This result was consistent with a previous study [22]. In our previous study, there were no significant differences in the association of age and gender between UC cases and controls. UC conventional risk factors such as status smoking carried 1.65-fold risk [23]. Smokers or people who had ever smoked and carried* TNF-α -*308 A/A genotype and the* IL-8* -251 T/T genotype had a higher risk of UC compared to nonsmokers. Gene-environment interactions are a potential way to identify individuals' risk. This study analyzed the combined effect of three major risk factors (high cumulative cigarette smoking, the* TNF*-*α* -308 A/A genotype, and the* IL-8* -251 T/T genotype) and found dose-response relationships for UC risk in study subjects with any one risk factor, any two risk factors, and all three risk factors.

Functional polymorphisms of inflammatory genes can affect cytokine production. A previous study reported that the* TNF-α -*308 G/A polymorphism contributes to carcinogenesis and that the variant A allele was associated with increased TNF-*α* expression [14]. A Korean study also reported that the* TNF-α -*308 G/A polymorphism was significantly associated with the tumor stage and grade of bladder cancer [24]. In this study, subjects who carried the A/A genotype of the* TNF-α -*308 G/A polymorphism had a significantly higher OR for UC than those with the G/G or G/A genotype. These results suggest that the* TNF-α -*308 G/A polymorphism might regulate angiogenesis, which plays a role in the invasion and metastasis of various tumors. However, another study did not report the same findings in urinary stone diseases or bladder cancer in Taiwan [25].

Compared to previous studies, differences in genotype frequencies may have been due to ethnic variations. The A/A genotype frequency of TNF-*α* in the case and control groups of this study was 2.23% and 0.67%, respectively, and the TNF-*α*A/A genotype carried a 3.56-fold risk of UC. A meta-analysis study showed that the A/A genotype frequency of TNF-*α* in cervical cancer cases and controls was 0.97%~7.33% and 0%~6.78%, respectively. The TNF-*α* A/A genotype significantly elevating risks of cervical cancer was found in a Caucasian population (OR: 2.09, 95% CI: 1.34~3.25) [26]. Although the TNF-*α* A/A genotype frequency was very low in Japanese (141 bladder cancer patients and 173 control subjects), the allelic frequency of patients (3.5%) was higher than that in controls (0.6%) [27]. A previous study indicated that cigarette smoking activates systemic inflammation and upregulates TNF-*α* expression in an animal model [8]. Another study also found that the circulating TNF-*α* level was higher in people who had ever smoked compared to people who had never smoked [17]. A study also showed that the A allele polymorphism of TNF-*α* -308 genotypes was associated with HCC risk in Taiwan males who were exposed to cigarette and alcohol [28]. In the present study, we observed a significant combined effect of cigarette smoking and the A/A genotype of the* TNF-α -*308 polymorphism on the OR (10.25) for UC. This finding suggests that the* TNF-α -*308 G/A polymorphism can modify the OR for UC, especially in people who had ever smoked.

IL-8 plays a critical role in inflammation. A common single-nucleotide polymorphism at the -251 position of the* IL-8* promoter region can influence its expression and may increase one's susceptibility to bladder cancer [29]. In the present study, subjects who carried the A/A genotype had a significantly decreased OR for UC. Previous studies explored the association between the* IL-8* -251 T/A polymorphism and various cancer risks, but the findings were inconsistent [29, 30]. Some researchers reported that the* IL-8* -251A allele has significantly higher promoter activity than the -251T allele [31]. However, another study reported that the* IL-8* -251T allele had significantly higher transcriptional activity than the -251A allele of the* IL-8* gene [32]. In addition, previous studies indicated that cigarette smoking causes chronic inflammation characterized by increased levels of cytokines, such as IL-8 [10, 11]. IL-8 plays a role in assisting cancer cells to eschew stress and induce apoptosis and is also involved in angiogenesis, tumor growth, and metastasis [33]. A meta-analysis showed that IL-8 -251A/T polymorphism has significantly elevated risks of cancer in Asian population [34]. It is possible that the increased IL-8 expression induced by cigarette smoking may modify the carcinogenesis of UC. In the present study, we found a significant joint effect of cigarette smoking and the T/T genotype of the -251 T/A polymorphism on the OR for UC (OR = 3.08). This suggests that the -251 T/A polymorphism can modify the UC risk, especially in people who have ever smoked.

Single-nucleotide polymorphisms of genes can increase disease susceptibilities via affect gene expression. Silico analysis is a novel computational approach to identify potential SNPs causing transcription factor binding affinities to change and influence regulatory functions of genes [35]. Since polymorphisms have been identified and their transition is considered to be an important enhancer of transcriptional activation associated with elevated levels genes expression, structural analysis study showed that TNF-*α* protein stability was impacted by amino acid residue substitutions of P84L (rs4645843) and A94T (rs1800620) [36]. The effect of IL-8 SNPs and protein structure in disease risk was also conducted by silico analysis [37]. Although the level of confidence is too high in some cases, it is still too low for clinical purpose, which is a main limitation of silico analysis programs [38].

Our study has some limitations. We only investigated one polymorphism located in the promoter region, which might not entirely account for the functions of the* TNF-α* and* IL-8* genes. More functional polymorphisms of the* TNF-α* and IL-8 genes should be included in future analyses with larger samples to validate our findings. Other proinflammatory cytokines such as NF-*κ*B also need to be further investigated. In addition, interactions of cigarette smoking and other cytokines on the UC risk should be explored in the future.

## 5. Conclusions

We found a dose-response relationship between the number of risk factors and the OR for UC. These findings suggest that individuals who carry high-risk genotypes, including the* TNF-α*-308 A/A genotype and the* IL-8* -251 T/T genotype, and who experience cumulative cigarette smoking have a significantly increased OR for UC in a dose-response manner.

## Figures and Tables

**Table 1:** Sociodemographic characteristics of 287 urothelial carcinoma cases and 574 matched noncancer controls, stratified by *TNF*-*α* and *IL-*8 genotypes.

Variable	TNF-*α*			IL-8		
	GG/GA	AA	*p* value	TT	TA/AA	*p* value
Age (years)	62.82 ± 13.56	59.18 ± 9.45	0.38	60.83 ± 13.55	65.59 ± 12.99	<0.0001
Gender						
Male	591 (69.53)	10 (90.91)	0.19	357 (69.89)	244 (69.71)	0.96
Female	259 (30.47)	1 (9.09)		154 (30.14)	106 (30.29)	
Educational level						
Illiterate or elementary school	229 (27.00)	1 (9.09)	0.41	124 (24.31)	106 (30.37)	0.05
Junior or senior high school	304 (35.85)	5 (45.45)		181 (35.49)	128 (36.68)	
College or university	315 (37.15)	5 (45.45)		205 (40.20)	115 (32.95)	
Cigarette smoking						
No	526 (61.88)	5 (45.45)	0.35	329 (64.38)	202 (57.71)	0.05
Yes	324 (38.12)	6 (54.55)		182 (35.62)	148 (42.29)	
Environmental tobacco exposure						
No	411 (51.31)	6 (54.55)	0.65	250 (51.98)	167 (50.45)	0.10
Yes	290 (36.20)	5 (45.45)		182 (37.84)	113 (34.14)	
Alcohol consumption						
No	497 (58.47)	5 (45.45)	0.68	297 (58.12)	205 (58.57)	0.11
Yes	167 (19.65)	3 (27.27)		111 (21.72)	59 (16.86)	
Occasional	186 (21.88)	3 (27.27)		103 (20.16)	86 (24.57)	

Educational level and environmental tobacco smoke were unavailable for two and 149 of the *TNF-α* GG/GA genotypes, respectively. Educational level and environmental tobacco smoke were unavailable for one and 79 of the *IL-*8 TT genotypes, respectively. Educational level and environmental tobacco smoke were unavailable for one and 70 of the *IL-*8 TA/AA genotypes, respectively.

**Table 2:** Dose-response relationship between cigarette smoking profiles and the odds ratio (OR) for urothelial carcinoma (UC).

	UC cases	Controls	Age/sex-adjusted OR (95% CI)	Multivariate-adjusted OR (95% CI)
Cigarette smoking (packs/day)				
0	152	379	1.00	1.00
0~1	108	169	1.69 (1.10~2.61)^*∗*^	1.90 (1.19~3.03)^*∗*^
>1	27	26	2.39 (1.62~3.54)^*∗*^	2.33 (1.52~3.55)^*∗*^
Cigarette smoking (years)				
0	153	379	1.00	1.00
0~31	49	101	1.46 (0.93~2.27)	1.49 (0.92~2.39)
>31	85	94	2.59 (1.74~3.86)^*∗*^	2.74 (1.77~4.24)^*∗*^
Cigarette smoking (pack years)				
0	153	379	1.00	1.00
0~18	35	81	1.19 (0.73~1.94)	1.30 (0.78~2.19)
>18	99	114	2.62 (1.80~3.81)^*∗*^	2.64 (1.75~3.99)^*∗*^

Multivariate ORs were adjusted for age, gender, educational level, and alcohol consumption. ^*∗*^*p* < 0.05. CI: confidence interval.

**Table 3:** Adjusted odds ratios (ORs) of urothelial carcinoma risk by cumulative cigarette exposure, the tumor necrosis factor (*TNF*)-*α* -308 A/A genotype, and the interleukin (*IL*)-8 -251 T/T genotype.

Risk factors	Age/sex-adjusted OR (95% CI)	Multivariate-adjusted OR (95% CI)
None	1.00	1.00
Any one risk factor	1.47 (0.99~2.19)	1.55 (1.03~2.35)^*∗*^
Any two risk factors	2.69 (1.63~4.43)^*∗*^	2.89 (1.70~4.93)^*∗*^
All three risk factors	3.61 (2.15~6.07)^*∗*^	3.77 (2.16~6.56)^*∗*^
	*p*_trend_ < 0.0001	*p*_trend_ < 0.0001

The three risk factors were cumulative cigarette exposure,the *TNF-α* -308 A/A genotype, and the* IL-*8 -251 T/T genotype. Multivariate ORs were adjusted for age, gender, educational level, and alcohol consumption. ^*∗*^*p* < 0.05. CI: confidence interval.

## Data Availability

The data used to support the findings of this study are included within the article.
